# HMGN5 promotes IL-6-induced epithelial-mesenchymal transition of bladder cancer by interacting with Hsp27

**DOI:** 10.18632/aging.103076

**Published:** 2020-04-21

**Authors:** Kun Yao, Leye He, Yu Gan, Jianye Liu, Jin Tang, Zhi Long, Jing Tan

**Affiliations:** 1 Department of Urology, The Third Xiangya Hospital of Central South University, Changsha 410013, Hunan, P.R. China; 2 Institute of Prostate Disease, Central South University, Changsha 410013, Hunan, P.R. China

**Keywords:** bladder cancer, high-mobility group nucleosome-binding domain 5 (HMGN5), heat shock protein 27 (Hsp27), epithelial-mesenchymal transition (EMT), signal transductor and activator of transcription 3 (STAT3)

## Abstract

Bladder cancer (BC) is one of the most common cancers worldwide, with a high rate of recurrence and poor outcomes. High-mobility group nucleosome-binding domain 5 (HMGN5) is overexpressed in many cancers and could cause carcinogenesis in BC. By protein-protein-interaction (PPI) analysis, we found that heat shock protein 27 (Hsp27), also a crucial functional factor in BC carcinogenesis, is significantly related to HMGN5. Hsp27 is required for IL-6-mediated EMT via STAT3/Twist signaling in prostate cancer. Here, we hypothesize that HMGN5 may interact with Hsp27 to affect IL-6-induced EMT and invasion in BC via STAT3 signaling. In the present study, we found that HMGN5 and Hsp27 are highly expressed in BC tissues and positively correlated with each other. HMGN5 interacts with Hsp27 *in vitro*, to modulate the cell invasion and EMT in BC. Moreover, HMGN5 could modulate IL-6-Hsp27-induced EMT and invasion in BC cells by regulating STAT3 phosphorylation and STAT3 targeting of the Twist promoter. HMGN5 interacts with Hsp27 to promote tumor growth in a human BC xenograft model in nude mice. In summary, HMGN5 interacts with Hsp27 to promote IL-6-induced EMT, therefore promoting invasion in BC and contributing to the progression of BC.

## INTRODUCTION

Bladder cancer is one of the most common malignant tumors globally with a high rate of recurrence and poor outcomes, as a result of relapse. Of all primary bladder cancers, approximately 90% are transitional cell carcinomas, 5% are squamous cell carcinomas, and 1–2% are adenocarcinomas [1, 2]. Developing an in-depth knowledge of the molecular and cellular mechanisms of tumorigenesis may provide new directions for bladder cancer therapy.

High-mobility group nucleosome-binding domain 5 (HMGN5) is one of the HMGNs found recently [3, 4]. Recent emerging research has reported that HMGN5 is overexpressed in many human cancers and that HMGN5 is related to carcinogenesis in a variety of cancer models [5], including bladder cancer [6]. In bladder cancer, HMGN5 silencing could block the PI3K/Akt signaling pathway, therefore improving the chemical sensitivity of human bladder cancer cells to cisplatin, whereby the viability and invasion of these cells could be inhibited *in vitro* and *in vivo* [7]. To study how HMGN5 exerts its effects on bladder cancer cell invasion and epithelial-mesenchymal transition (EMT), a crucial issue during cancer metastasis, we performed protein-protein-interaction (PPI) analysis to search for factors related to HMGN5. Of the selected candidates, heat shock protein 27 (Hsp27), another crucial functional factor in bladder cancer carcinogenesis [8, 9], drew our attention.

As molecular chaperones, Hsps exert their biological effects by holding protein and serving as the folding of the newly synthesized protein to maintain cellular homeostasis [10]. A small Hsp, Hsp27 responds to heat shock and other cellular stressors such as cancers [11, 12]. Additionally, Hsp27 expression has been reported to be significantly associated with bladder cancer invasiveness and prognosis in bladder cancer patients [13, 14]. Based on its properties, Hsp27 has been considered a therapeutic target for many malignant tumors. For example, OGX-427 is a second-generation antisense oligonucleotide sequence designed to bind to Hsp27 mRNA. It can induce apoptosis, improve the sensitivity to chemotherapy drugs and suppress cellular proliferation, indicating its clinical potential in bladder cancer therapy [14–16]. In prostate cancer, silencing Hsp27 can decrease IL-6-dependent STAT3 phosphorylation, nuclear translocation, and STAT3 targeting of the Twist promoter, indicating that Hsp27 is required for IL-6-mediated EMT via regulation of STAT3/Twist signaling [17]. Thus, we hypothesize that HMGN5 may interact with Hsp27 to affect IL-6-induced EMT and invasion in bladder cancer by modulating STAT3 phosphorylation and STAT3 targeting of the Twist promoter.

Here, the expression of HMGN5 and Hsp27 and their correlation in tissue samples were evaluated. The predicted interaction between the HMGN5 and Hsp27 proteins was examined. Next, the dynamic effects of HMGN5 and Hsp27 on IL-6-independent- and IL-6-dependent EMT and invasion in bladder cancer cells were evaluated. Furthermore, we examined whether HMGN5 could modulate IL-6-Hsp27-induced STAT3 phosphorylation and STAT3 targeting of Twist promoter. Finally, the dynamic effects of HMGN5 and Hsp27 on tumor growth were investigated in nude mice. In summary, we provide a novel mechanism and experimental evidence showing how the interaction between HMGN5 and Hsp27 affects bladder cancer cell invasion and EMT with or without IL-6.

## RESULTS

### Expression and correlation of HMGN5 and Hsp27 in tissue samples

**Figure 1:**
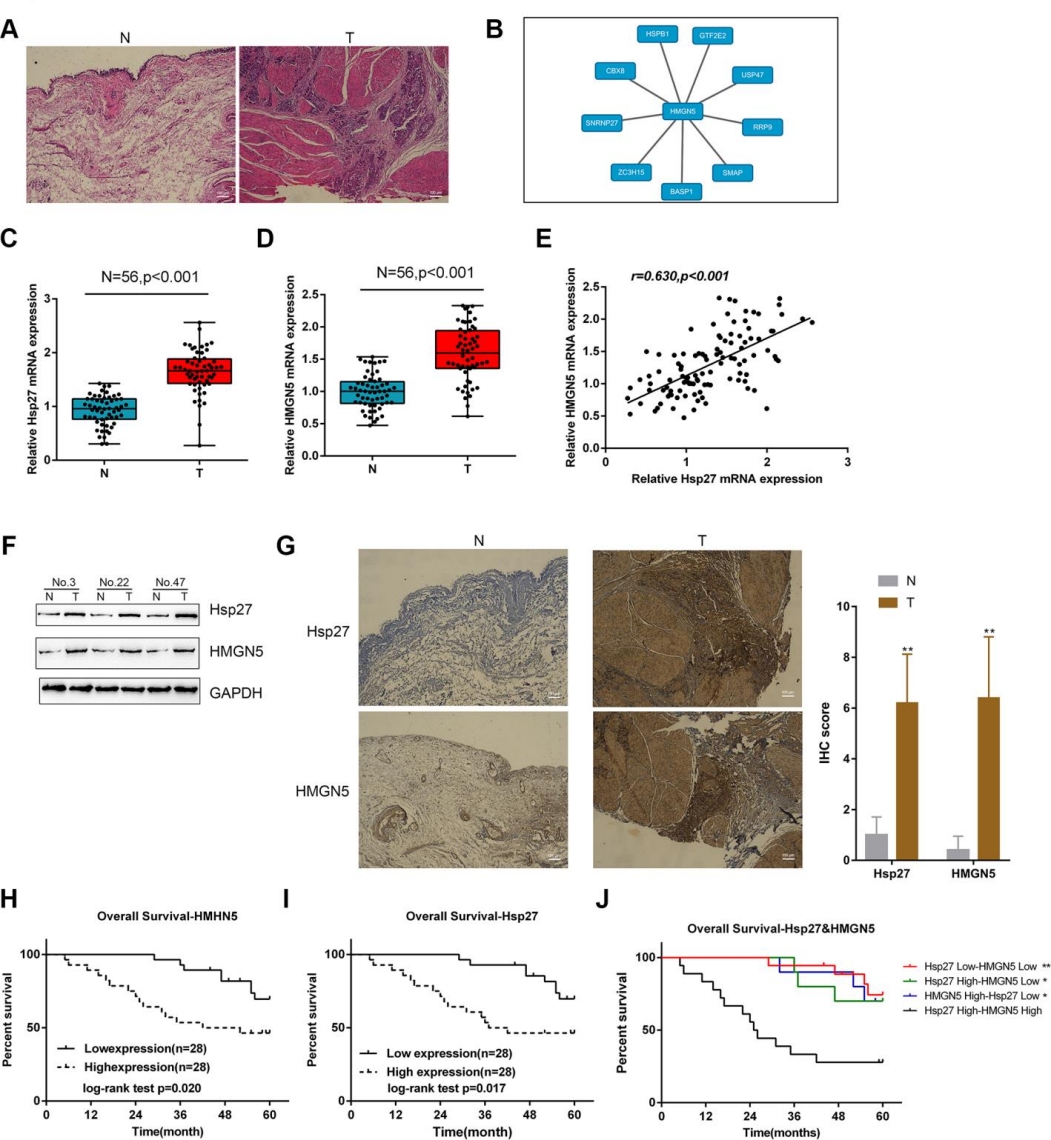
**Expression and correlation of HMGN5 and Hsp27 in tissue samples.** (**A**) Pathological characteristics of normal and bladder cancer tissues assessed by HE staining. (**B**) Protein-protein-interaction analysis showing proteins related to HMGN5 based on data from BioGRID (https://thebiogrid.org/). (**C**, **D**) The expression of Hsp27 and HMGN5 in 56 paired normal and bladder cancer tissues examined by qPCR. (**E**) The correlation of Hsp27 and HMGN5 mRNA expression in tissue samples analyzed by Pearson’s correlation analysis. (**F**) The protein levels of Hsp27 and HMGN5 in tissue samples were examined by immunoblotting. (**G**) The protein levels and localization of Hsp27 and HMGN5 in tissue samples were examined by IHC staining. (**H**–**J**) The association of Hsp27 and HMGN5 expression with overall survival was analyzed by Kaplan-Meier overall survival analysis.

The obtained tissue samples were examined for pathological characteristics by HE staining (Figure 1A). Proteins related to HMGN5 were analyzed by BioGRID (Biological General Repository for Interaction Datasets), and a total of 9 proteins were identified (Figure 1B). Among them, Hsp27 mRNA expression was the most upregulated in 10 bladder cancer tissues when compared with noncancerous tissue (Supplementary Figure 1). Moreover, Hsp27 was selected for further experiments because it is one of the Hsps overexpressed in bladder cancer and protects tumor cells from therapeutic stressors [9]. Similar to previous investigations, Hsp27 and HMGN5 mRNA expression was remarkably upregulated in 56 cases bladder cancer tissues (Figure 1C, 1D), and the expression levels were positively correlated with each other (Figure 1E). As demonstrated by immunoblotting and IHC staining, the protein levels of HMGN5 and Hsp27 were higher in bladder cancer tissues (Figure 1F, 1G).

Next, we analyzed the correlation of the overall survival rate with HMGN5 and Hsp27 levels. Bladder cancer patients were divided into two groups based on the expression of Hsp27 or HMGN5 using the med ian expression value as the cutoff. As shown in Figure 1H and I, lower Hsp27 or lower HMGN5 expression was related to a higher rate of overall survival in bladder cancer patients. Moreover, the group with high coexpression of HMGN5 and Hsp27 group showed the lowest overall survival rate (Figure 1J). These data suggest that HMGN5 may interact with Hsp27 to play a role in bladder cancer progression.

### HMGN5 interacts with Hsp27

**Figure 2:**
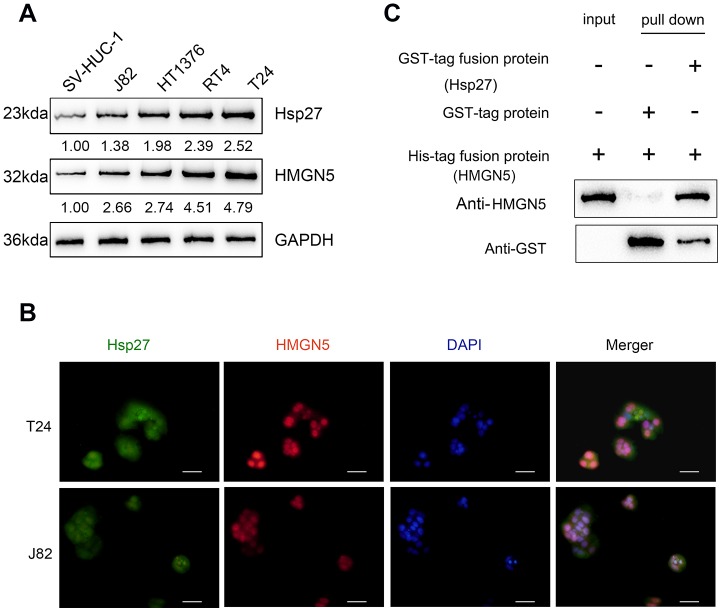
**HMGN5 interacts with Hsp27 *in vivo* and *in vitro.*** (**A**) The protein levels of Hsp27 and HMGN5 in bladder cancer cell lines examined by immunoblotting. (**B**) The protein levels and localization of Hsp27 and HMGN5 in T24 and J82 cells examined by IF staining. (**C**) *In vitro* GST pull-down assays were used to examine the interaction between HMGN5 and Hsp27.

To validate the predicted interaction between HMGN5 and Hsp27, we first examined their protein levels in four bladder cancer cell lines, J82, HT1376, RT4, and T24 by immunoblotting. Figure 2A shows that the protein levels of both HMGN5 and Hsp27 in the four BC cell lines are all higher than those in the normal cell line, SV-HUC-1. IF staining images showed the fluorescence intensity representing HMGN5 (red) and Hsp27 (green) expression (Figure 2B). The merged image also revealed that HMGN5 and Hsp27 expression might be colocalized in bladder cancer cells (Figure 2B). Moreover, we conducted an *in vitro* GST pull-down assay to validate the predicted interaction between HMGN5 and Hsp27. We overexpressed GST-tagged Hsp27 in bacteria and incubated them with His-tagged HMGN5 at 4 °C for 4 h. HMGN5 was captured by Ni-NTA resin, and Hsp27 was detected in the precipitated product by immunoblotting, suggesting that HMGN5 could interact with Hsp27 (Figure 2C).

### Overexpression of HMGN5 and Hsp27 promotes bladder cancer cell invasion and EMT

**Figure 3:**
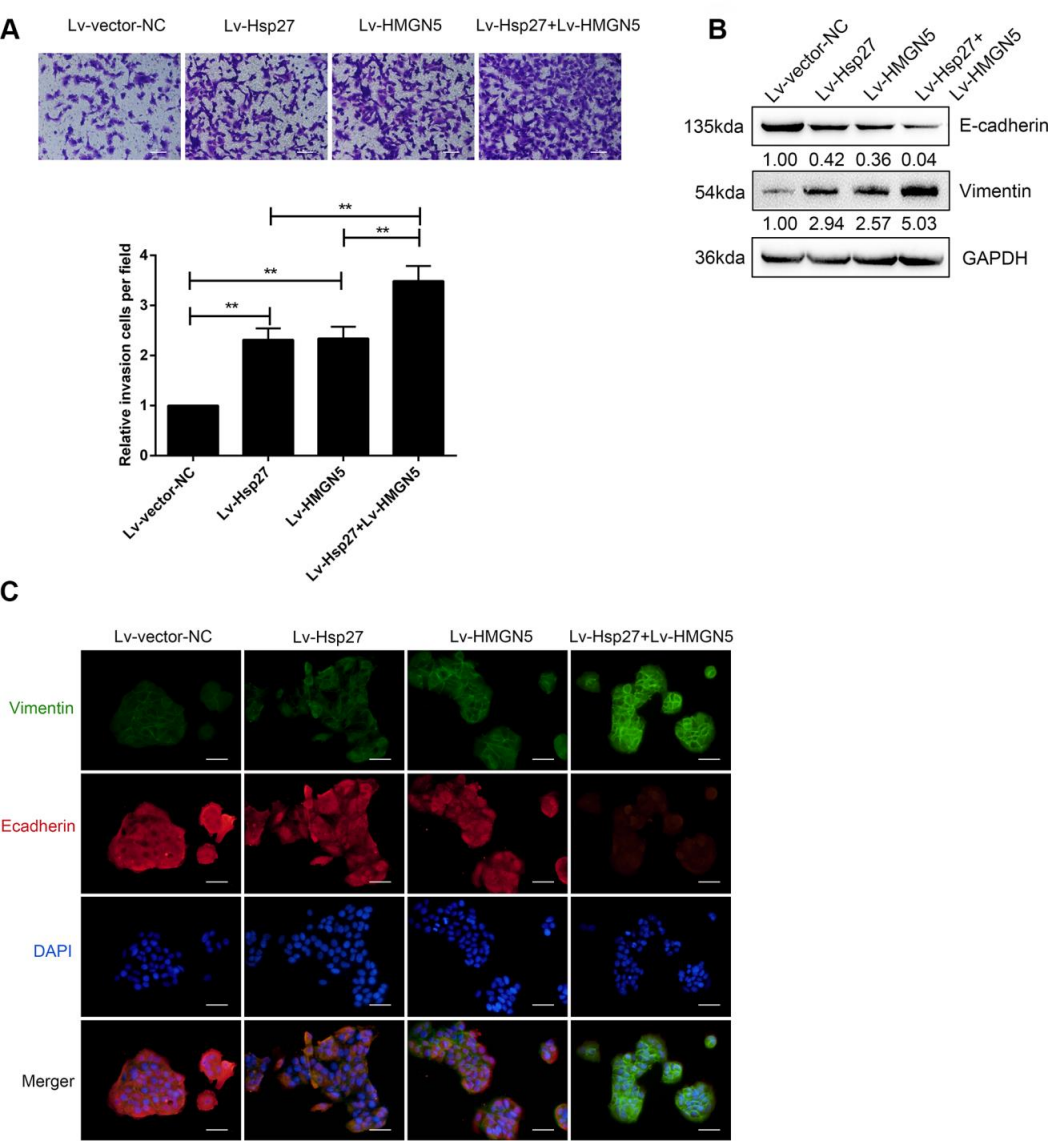
**HMGN5 interacts with Hsp27 to modulate bladder cancer cell invasion and EMT.** (**A**) J82 cells were transduced with Lv-HMGN5 or Lv-Hsp27, separately, or cotransduced with Lv-HMGN5 and Lv-Hsp27, and examined for cell invasion by Transwell assays. (**B**) The protein levels of E-cadherin and Vimentin, as demonstrated by immunoblotting, (**C**) The protein levels of E-cadherin and Vimentin by IF staining. The data are presented as the mean ± SD of three independent experiments. **P<0.01.

We confirmed the interaction between HMGN5 and Hsp27; then, we examined the effect of HMGN5 and Hsp27 overexpression on bladder cancer cell invasion and EMT. HMGN5 and Hsp27 overexpression was achieved in J82 cells, as confirmed by immunoblotting (Supplementary Figure 2A, 2B). Transwell assays revealed that HMGN5 or Hsp27 overexpression alone could significantly promote bladder cancer cell invasion (Figure 3A) and decrease E-cadherin expression and increase Vimentin expression (Figure 3B, 3C). More importantly, Hsp27 overexpression further enhanced the promoting effect of HMGN5 overexpression (Figure 3A–3C), suggesting that HMGN5 interacts with Hsp27 to modulate bladder cancer cell invasion and EMT.

### Silencing of HMGN5 and Hsp27 inhibits bladder cancer cell invasion and EMT

**Figure 4:**
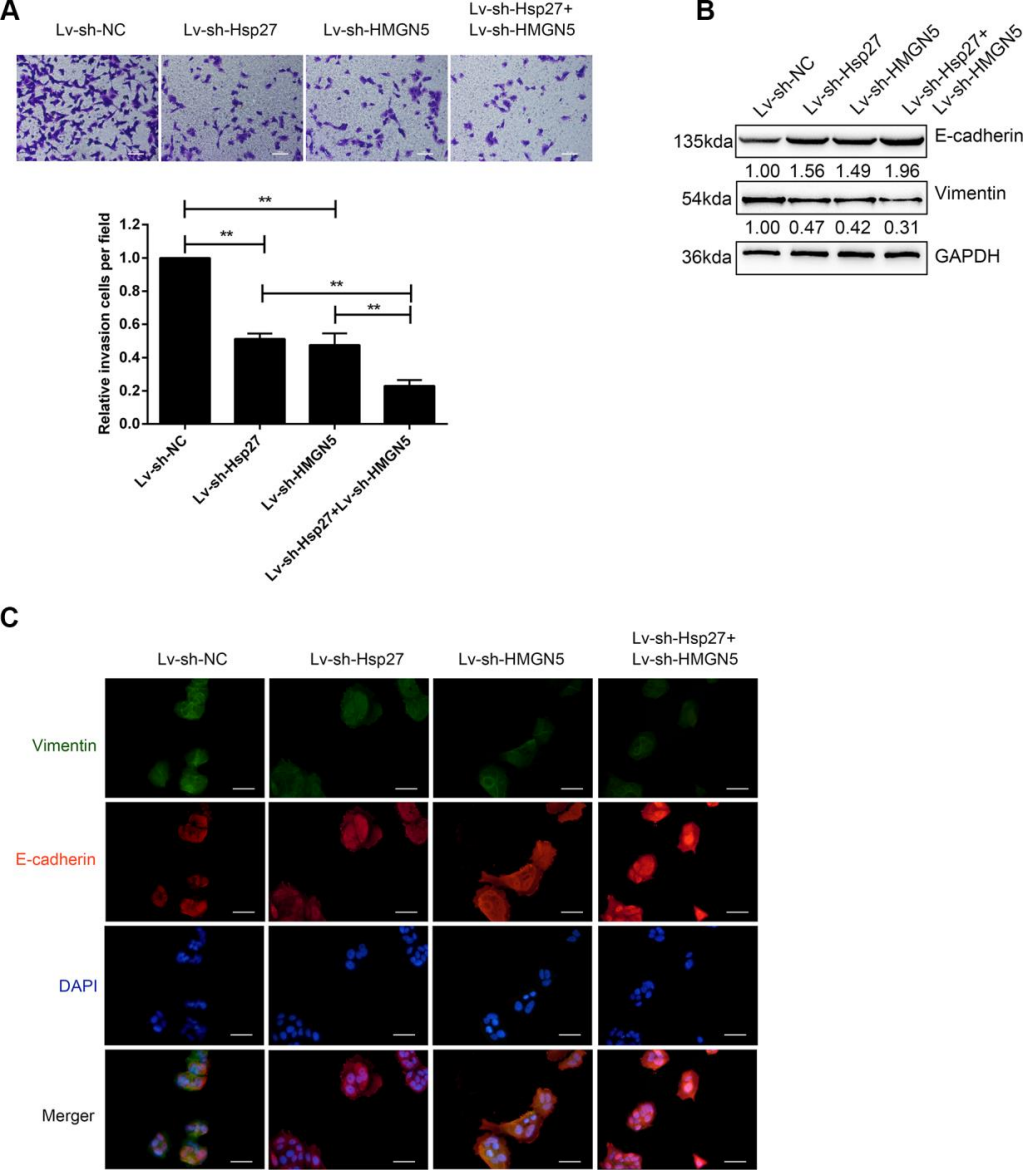
**Silencing of HMGN5 and Hsp27 inhibits bladder cancer cell invasion and EMT.** (**A**) T24 cells were transduced with Lv-sh-HMGN5 or Lv-sh-Hsp27, separately, or cotransduced with Lv-sh-HMGN5 and Lv-sh-Hsp27, and examined for cell invasion by Transwell assays. (**B**) The protein levels of E-cadherin and Vimentin by immunoblotting. (**C**) The protein levels of E-cadherin and Vimentin by IF staining. The data are presented as the mean ± SD of three independent experiments. **P<0.01.

To further validate the dynamic effects of HMGN5 and Hsp27 on bladder cancer cells, we examined how HMGN5 and Hsp27 silencing affected bladder cancer cell invasion and EMT. T24 cells were infected with Lv-sh-HMGN5 or Lv-sh-Hsp27 alone to silence HMGN5 or Hsp27, as confirmed by immunoblotting (Supplementary Figure 2A, 2B). Next, T24 cells were coinfected with Lv-sh-HMGN5 and Lv-sh-Hsp27 and examined for cell invasion and EMT. In contrast to HMGN5 or Hsp27 overexpression, HMGN5 or Hsp27 silencing significantly inhibited bladder cancer cell invasion (Figure 4A) and increased the E-cadherin expression and decreased Vimentin expression (Figure 4B, 4C). Similarly, the effect of HMGN5 silencing could be enhanced by Hsp27 silence (Figure 4A–4C), further suggesting that HMGN5 interacts with Hsp27 to modulate bladder cancer cell invasion and EMT.

### HMGN5 is involved in IL-6-Hsp27-induced cell invasion and EMT in bladder cancer cells

**Figure 5:**
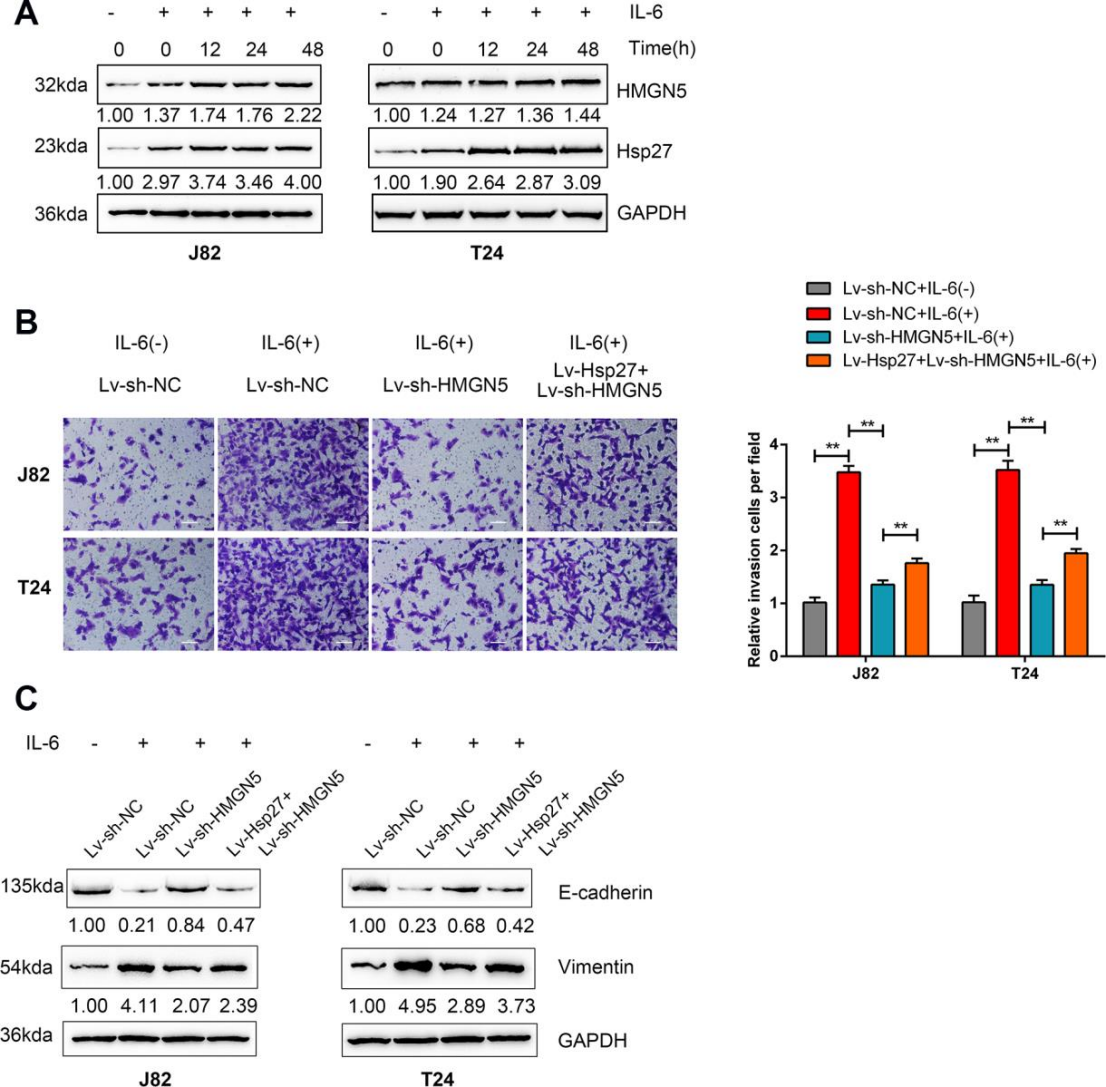
**HMGN5 is involved in IL-6-Hsp27-induced cell invasion and EMT in bladder cancer cells.** (**A**) T24 cells were serum starved overnight and treated with 50 ng/mL IL-6 for 0 to 48 h (0, 6, 12, 24, 48 h) and examined for the protein levels of HMGN5 and Hsp27 by immunoblotting. (**B**) T24 cells were cotransduced with Lv-Hsp27 and Lv-sh-HMGN5, serum starved overnight, treated with 50 ng/mL IL-6 for 24 h and examined for cell invasion by Transwell assays, and (**C**) the protein levels of E-cadherin and Vimentin were determined by immunoblotting, compared to those in Lv-Vector-transduced cells without IL-6 treatment. The data are presented as the mean ± SD of three independent experiments. **P<0.01.

In prostate cancer, Hsp27 is involved in IL-6-mediated EMT [17]. Here, we investigated whether HMGN5 participates in IL-6-Hsp27-induced EMT in bladder cancer. After 24 h of serum starvation, the protein levels of HMGN5 and Hsp27 were induced by IL-6 stimulation in both J82 and T24 cells in a time-dependent manner (Figure 5A).

Next, J82 and T24 cells were coinfected with Lv-Hsp27 and Lv-sh-HMGN5 and examined for cell invasion and EMT markers with or without 50 ng/mL IL-6 stimulation. As shown in Figure 5B and 5C, cell invasion and EMT in both J82 and T24 cell lines could be induced by IL-6 stimulation but inhibited by HMGN5 knockdown. However, the HMGN5 knockdown-mediated suppression of cell invasion and EMT could be partially rescued by Hsp27 overexpression. These findings suggest that HMGN5 is involved in IL-6-Hsp27-induced cell invasion and EMT in bladder cancer cells.

### HMGN5/Hsp27 modulates IL-6-induced STAT3 phosphorylation and Twist promoter activity

**Figure 6:**
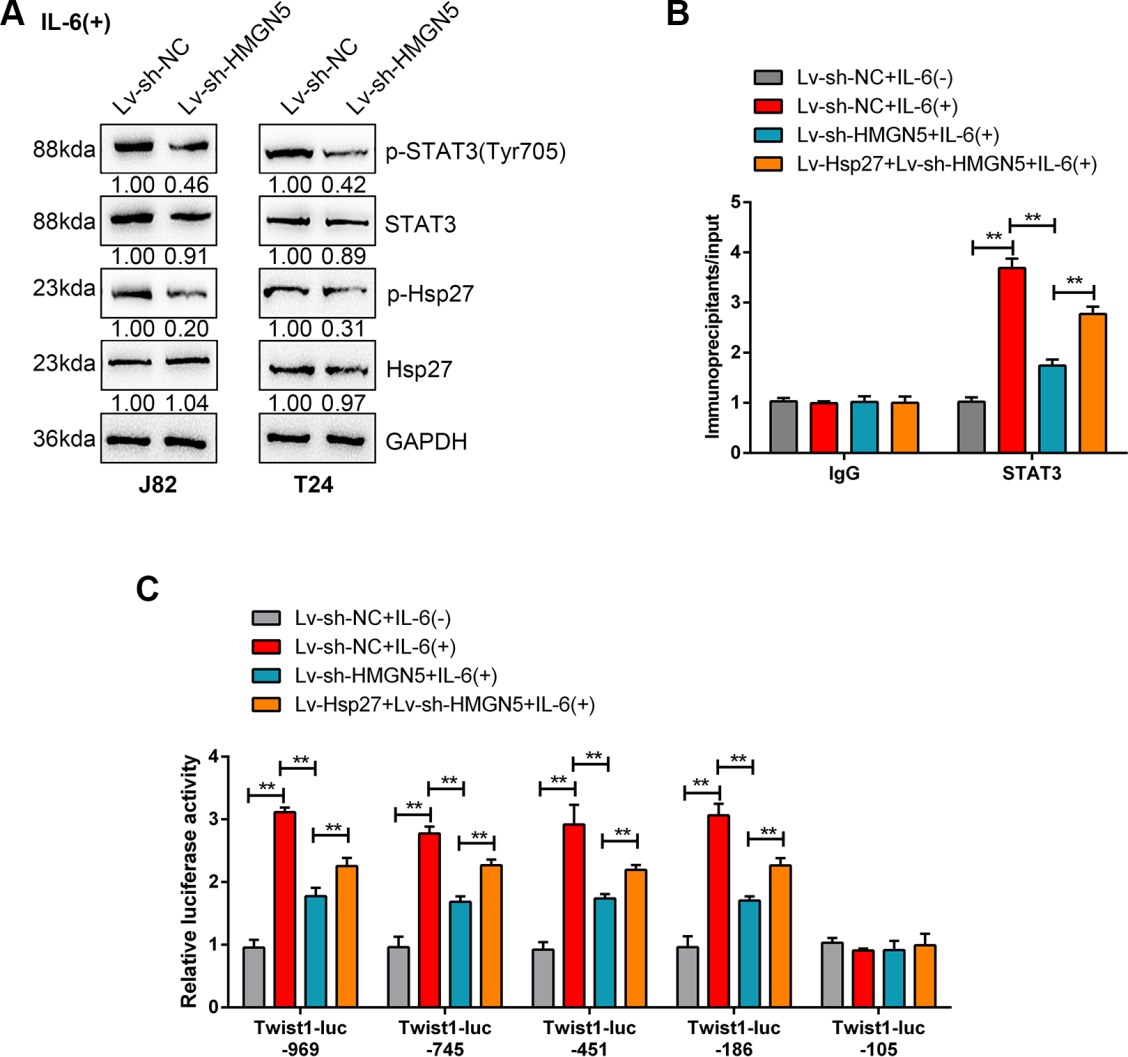
**HMGN5/Hsp27 modulates IL-6-induced STAT3 phosphorylation and Twist promoter activity.** (**A**) J82 and T24 cells were transduced with Lv-sh-HMGN5 under IL-6 stimulation and examined for the protein levels of p-STAT3, STAT3, p-Hsp27, and Hsp27. (**B**) ChIP assays were conducted on nuclear extracts from T24 cells cotransduced with Lv-sh-HMGN5 and Lv-Hsp27 and treated with or without IL-6, after which immunoprecipitations were conducted with anti-IgG or anti-STAT3 and Protein G agarose. Real-time PCR was conducted using immunoprecipitated DNAs, soluble chromatin, and specific primer pairs for Twist. The results of immunoprecipitated samples were corrected for the results of the corresponding soluble chromatin samples. (**C**) T24 cells were treated as described above and were cotransfected with 0.5 μg/mL of Twist-Luc plasmid and 0.05 μg/mL pRL-TK and treated with or without 50 ng/mL IL-6 for 6 h before lysis. The luciferase activity of Twist-Luc–969 alone was set as 1. The data are presented as the mean ± SD of three independent experiments. **P<0.01.

It has been reported that STAT3-mediated Twist transcription is involved in IL-6/Hsp27-mediated EMT via STAT3 targeting of the Twist promoter in prostate cancer [17]. Next, we validated whether HMGN5/Hsp27 could modulate IL-6-induced STAT3 phosphorylation and Twist promoter activity. HMGN5 silencing significantly decreased the phosphorylation of Hsp27 and STAT3 induced by IL-6 stimulation in J82 and T24 cells (Figure 6A).

We performed ChIP assays to examine the dynamic effects of HMGN5 silencing and Hsp27 overexpression on STAT3 targeting of the Twist promoter [18], with or without IL-6 stimulation. The inducing effects of IL-6 on STAT3 targeting of Twist promoter DNA could be significantly attenuated by HMGN5 silencing but partially rescued by Hsp27 overexpression (Figure 6B). Additionally, we performed luciferase reporter analysis to investigate the dynamic effect of HMGN5 silencing and Hsp27 overexpression on IL-6-induced Twist transcriptional activity. As described by Cheng et al., we used serial truncations of the human Twist promoter to analyze Twist transcriptional activity [18] with or without IL-6. Consistent with the previous study, in each assessment of Twist truncation, promoter activation was significantly induced by IL-6, which could be remarkably suppressed by HMGN5 knockdown but significantly rescued by Hsp27 overexpression (Figure 6C). These findings suggest that HMGN5 interacts with Hsp27 to modulate IL-6-induced STAT3 targeting of Twist promoter and the activation of the Twist transcription.

### HMGN5 interacts with Hsp27 to promote tumor growth in nude mouse model

**Figure 7:**
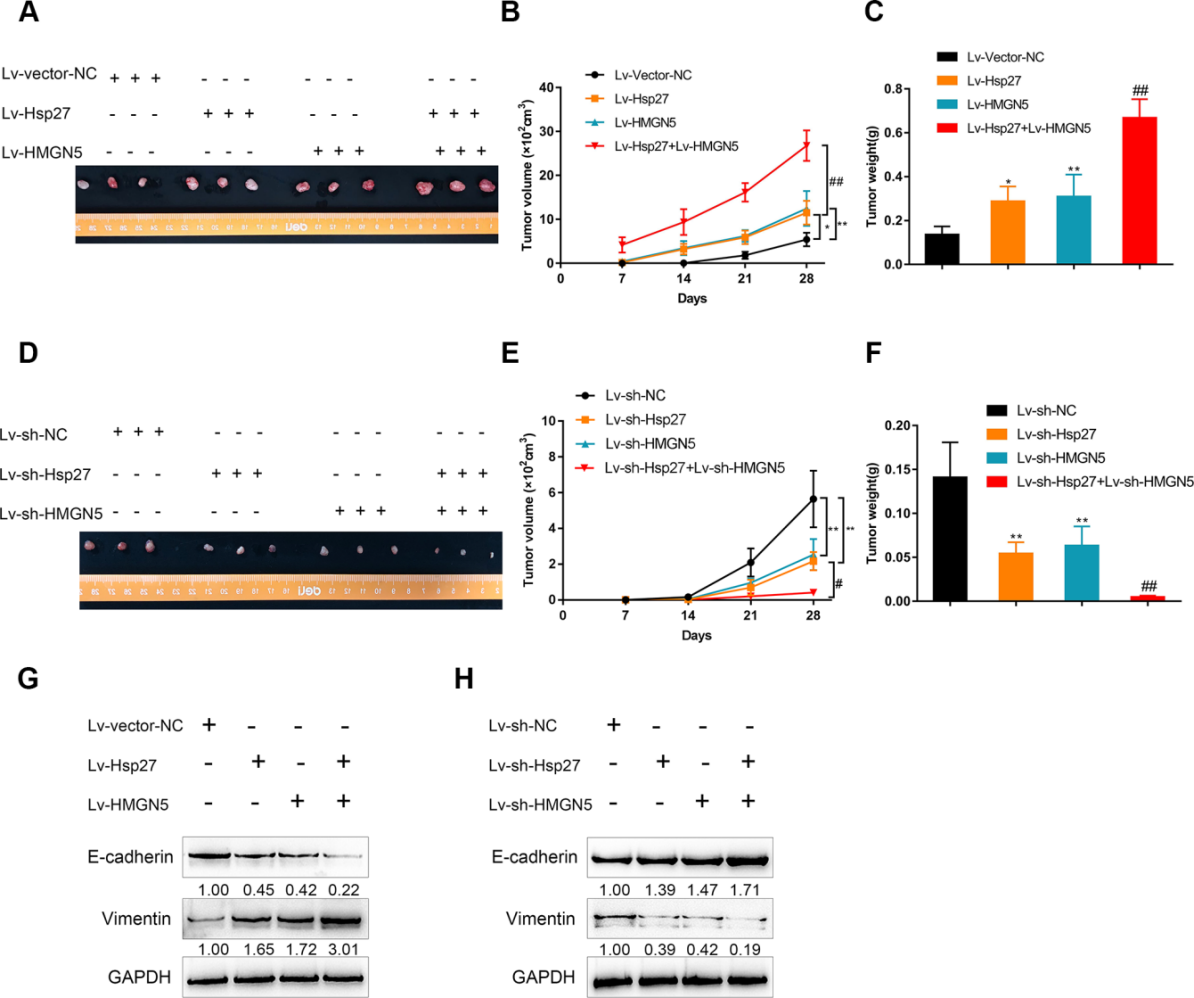
**HMGN5 interacts with Hsp27 to modulate tumor growth in a nude mouse model.** The appearance (**A**), tumor volume (**B**), and tumor weight (**C**) of tumors derived from Hsp27 and/or HMGN5-overexpressing J82 cells. The appearance (**D**), tumor volume (**E**), and tumor weight (**F**) of tumors derived from Hsp27 and/or HMGN5 silenced J82 cells. (**G**) The protein levels of E-cadherin and Vimentin in tumors derived from Hsp27 and/or HMGN5 overexpressing J82 cells. (**H**) The protein levels of E-cadherin and Vimentin in tumors derived from Hsp27 and/or HMGN5 silenced J82 cells. The data are presented as the mean ± SD of three independent experiments. *P<0.05, **P<0.01, ^##^*P*<0.01.

To further validate the above *in vitro* findings, we established a xenograft model of human bladder cancer in nude mice using HMGN5- and/or Hsp27-overexpressing or silenced J82 cells. Figure 7A shows the appearance of tumors derived from J82 cells with HMGN5- and/or Hsp27-overexpression. As shown in Figure 7B, 7C, HMGN5 or Hsp27 overexpression alone was sufficient to increase the tumor volume and tumor weight, which was further increased by the combination of HMGN5 and Hsp27 overexpression. In contrast, knockdown of Hsp27 or HMGN5 alone clearly decreased restrained the tumor volume and tumor weight, and the inhibitory effect on tumor volume and tumor weight was amplified in the group with combined HMGN5 and Hsp27 silencing (Figure 7D–7F). The protein levels of EMT markers could be increased by either HMGN5 or Hsp27 overexpression, and further increased by the combination of HMGN5 and Hsp27 overexpression (Figure 7G). Conversely, the EMT marker protein levels were inhibited by either HMGN5 or Hsp27 silencing, and further inhibited by the combination of HMGN5 and Hsp27 silencing (Figure 7H). These findings suggest that HMGN5 interacts with Hsp27 to promote tumor growth in a nude mouse model.

## DISCUSSION

Here, we demonstrate that HMGN5 and Hsp27 are highly-expressed in bladder cancer tissues and positively correlated with each other. HMGN5 interacts with Hsp27, *in vitro* and *in vivo*, to modulate cell invasion and EMT in bladder cancer cells. Moreover, HMGN5 could modulate IL-6-Hsp27-induced EMT and invasion in bladder cancer cells by regulating STAT3 phosphorylation and STAT3-mediated Twist transcription. HMGN5 interacts with Hsp27 to promote tumor growth in a bladder cancer xenograft model in nude mice.

HMGN5 and Hsp27 have both been regarded as oncogenic factors in bladder cancer. HMGN5 is highly expressed in human bladder cancer, thus promoting bladder cancer cell proliferation and invasion [6, 19]. Similarly, the expression of Hsp27 is upregulated in bladder cancer [13, 15, 20–22]. In the present study, we observed consistent results that, HMGN5 and Hsp27 mRNA and protein levels were increased in bladder cancer tissues. Moreover, as predicted by BioGRID, HMGN5 could interact with Hsp27 *in vivo* and *in vitro*, as revealed by GST pull-down and IF staining.

Regarding the molecular and cellular functions of the interaction between HMGN5 and Hsp27, the dynamic effects of these two factors on bladder cancer cell EMT and invasion were examined. EMT is a complicated process, encompassing changes in the cytoskeleton and a decrease in E-cadherin expression [23]. Both the loss of epithelial markers and gain of mesenchymal markers have been revealed in many cancers, including in bladder cancer [24–28]. During EMT in bladder cancer, the loss of E-cadherin occurs frequently [29]. Furthermore, mesenchymal markers, Twist and Vimentin, are related to the stage and grade of bladder cancer and could strongly affect the progression and metastasis of bladder cancer [27, 30, 31]. Herein, HMGN5 or Hsp27 overexpression significantly promoted EMT by increasing E-cadherin and decreasing Vimentin, and promoted the invasion of bladder cancer cells. When combined, the effect of HMGN5 or Hsp27 overexpression was enhanced. Conversely, HMGN5 and Hsp27 silencing synergistically inhibited EMT and invasion in bladder cancer cells. Thus, HMGN5 could interact with Hsp27 to promote bladder cancer EMT, therefore enhancing bladder cancer cell invasion.

Chronic inflammation and increased inflammatory mediators could result in tumor progression, invasion, and angiogenesis [32, 33]. Proinflammatory cytokines, such as IL-6, IL-8, and TNF-α, play critical roles in the pathogenesis of bladder cancer [34]. Furthermore, as confirmed by the previous study, persistent STAT3 activation could maintain constitutive NF-κB activity, thus proving the relationship between oncogenic signaling pathways in the inflammatory microenvironment [35]. IL-6 is considered as not only a primary activator of STAT3 signaling pathways, but also the central cytokine that could affect the human inflammatory response [36, 37]. Moreover, as confirmed by the present study, IL-6 induced HMGN5 and Hsp27 protein levels in a time-dependent manner. HMGN5 silencing attenuated IL-6-induced bladder cancer EMT and bladder cancer cell invasion. Regarding the cellular mechanism, IL-6 could induce the phosphorylation of STAT3 [17], which could be partially attenuated by HMGN5 silencing. Moreover, IL-6-induced STAT3 targeting of the Twist promoter could also be suppressed by HMGN5 silencing. More importantly, the above-described effects of HMGN5 silencing could all be significantly reversed by Hsp27 overexpression, suggesting that HMGN5 is involved in IL-6-Hsp27-induced STAT3 phosphorylation and STAT3 targeting of the Twist promoter, therefore modulating IL-6-induced bladder cancer EMT and bladder cancer cell invasion.

## CONCLUSIONS

HMGN5 interacts with Hsp27 to modulate IL-6-independent- or IL-6-dependent EMT and cell invasion in bladder cancer cells. Upon IL-6 stimulation, HMGN5/Hsp27 modulates bladder cancer EMT and bladder cancer cell invasion via the STAT3/Twist signaling pathway.

## MATERIALS AND METHODS

### Clinical tissue samples

A total of 56 paired bladder urothelial carcinoma (transitional cell carcinoma) and noncancerous clinical tissue (2 cm from the tumor edge) samples were collected from patients receiving surgical resection in The Third Xiangya Hospital of Central South University with the approval of Central South University Ethics Review Committees. Tissues were immediately fixed in formalin or stored at −80°C. Written consents were obtained from all patients. The clinical characteristics of the patients are listed in Supplementary Table 1. The Univariate and multivariate analysis for factors related to overall survival were shown in Supplementary Table 2.

### Cell lines, cell culture, and cell transfection

Four bladder cancer cell lines, J82 (ATCC^®^ HTB-1^™^), HT-1376 (ATCC^®^ CRL-1472^™^), RT4 (ATCC^®^ HTB-2^™^), and T24 (ATCC^®^ HTB-4^™^) and a normal human urothelial cell line, SV-HUC-1 (ATCC® CRL-9520™), was obtained from ATCC (Manassas, VA, USA). J82 and HT-1376 cell lines were cultured in Eagle's Minimum Essential Medium (EMEM, Sigma, St. Louis, MO, USA) supplemented with 10% fetal bovine serum (FBS) (Sigma). RT4 and T24 cells were cultured in McCoy's 5a Modified Medium (ATCC, Catalog No. 30-2007) supplemented with 10% FBS. All cells were cultured at 37°C with 5% CO_2_.

### Lentivirus production, titration, and infection

To generate HMGN5 or Hsp27 silenced or overexpressed or control lentivirus, we cotransfected the plasmids encoding HMGN5/Hsp27 or HMGN5 shRNA/Hsp27 shRNA or the control scrambled sequence into 293T cells together with the plasmids pHelper1.0 and pHelper2.0 (GeneChem) for virus packaging using Lipo2000 (Invitrogen) following the manufacturer’s protocols. Then, lentivirus was harvested from the supernatants by ultracentrifugation and examined for the viral titer determination.

For lentiviral infection, cells at 40-50% confluence were incubated overnight. Then, replace the culture medium was replaced with 5ml/well viral supernatant at the appropriate titer, and the cells were incubated at 37°C for 10 h. After that, the viral supernatant was replaced with fresh media. Forty-eight hours later, the infected cells were selected with 2mg/ml puromycin. Five days later, the HMGN5 inhibition efficiency was examined by immunoblotting.

### Hematoxylin and eosin (HE) staining

Collected bladder urothelial carcinoma tissue specimens were fixed in 10% formalin overnight and then processed for paraffin embedding and sectioning. Sections of 4 μm were deparaffinized, rehydrated, and then stained using HE staining kit (Beyotime, Shanghai, China) according to the protocols.

### Immunohistochemistry (IHC) staining

Hsp27 and HMGN5 protein levels and localization were examined by IHC. Three paired of carcinoma and noncancerous tissue sections were randomly selected and fixed in acetone for 10 min at −20°C, permeabilized with 0.2% Triton (Sigma) for 10 min at room temperature, incubated with a blocking solution (3.75% BSA/5% goat serum, Zymed, Carlsbad, CA, USA) for 30 min, and incubated for 2 h with anti-Hsp27 (dilution 1:200, ab2790, Abcam, Cambridge, CA, USA) or anti-HMGN5 (dilution 1:200, SAB4301006, Sigma-Aldrich, St. Louis, MO, USA) antibody. All sections were incubated with the appropriate HRP-labeled secondary antibodies (dilution 1:100), then incubated with freshly prepared DAB reagent (Beyotime, China), and subsequently counterstained with hematoxylin (Beyotime, China). The sections were observed under a light microscope (Olympus, Tokyo, Japan). Tissue specimens were scored according to the intensity of the staining and the number of positive cells. The staining intensity was graded as 0 (no color), 1 (light yellow), 2 (light brown), or 3 (brown), and the number of positive cells was graded as 0 (<5%), 1 (5-25%), 2 (25-50%), 3 (51-75%), or 4 (>75%). The two grades were added together as the total score. IHC scores of Hsp27 and HMGN5 protein expression are is the average score of the three randomly selected tissue samples.

### Immunoblotting

The protein levels of Hsp27, p-Hsp27, HMGN5, E-cadherin, Vimentin, STAT3, and p-STAT3 were determined by immunoblotting. After lysing the target cells with RIPA buffer supplemented with 1% PMSF, we extracted the proteins and determined the protein concentration using the bicinchoninic acid (BCA) protein assay kit (Beyotime Institute of Biotechnology, China). An SDS-PAGE minigel was used for protein separation and the proteins were then transferred onto a PVDF membrane, which was then probed with the following antibodies (dilution 1:1000) : anti-Hsp27 (ab2790, Abcam), anti-p-Hsp27 (ab5594, Abcam), anti-HMGN5 (ab56031, Abcam), anti-E-cadherin (ab1416, Abcam), anti-Vimentin (ab8978, Abcam), anti-STAT3 (ab68153, Abcam), anti-p-STAT3 (ab76315, Tyr705, Abcam), and anti-GAPDH (ab8245, Abcam) at 4°C overnight followed by the incubation with the HRP-conjugated secondary antibody (dilution 1:5000). GAPDH was used as a reference protein. An ECL substrates (Millipore, MA, USA) was used to visualize the signals and ImageJ software (NIH) was used to perform the band intensity analysis.

### GST pull-down assay

The HMGN5-Hsp27 interaction was validated by GST pull-down analysis. GST-tagged protein and His-tagged protein were generated and purified following the method described previously [38]. The GST-fused protein was incubated with prepared glutathione Sepharose beads (GE Healthcare, Glutathione Sepharose 4B, 17-0756-01). Thirty minutes later, the beads were collected, washed, and incubated with the input proteins (0.1 mg/mL) or His-fused protein dissolved in the reaction buffer (20 mM Tris, 100 mM NaCl, 1 mM DTT and 1 mM EDTA) for 30 min. The supernatant was discarded and the beads together with the target proteins were washed with the reaction buffer four times. After that, the target proteins were washed with 10% SDS and the eluate was analyzed and detected by SDS-PAGE and immunoblotting.

### Immunofluorescence (IF) staining

Cells were fixed in 4% paraformaldehyde for 30 min and then permeabilized with 0.2% Triton X-100 for 15 min. After blocking with 1% BSA in PBS for 2 h, the cells were then incubated with specific antibodies (dilution 1:200) overnight at 4°C followed by incubation with TRITC or FITC-conjugated secondary antibody (dilution 1: 500, Beyotime, China) for 1 h in the dark. Nuclear staining was performed using DAPI (Beyotime, China). A fluorescence microscope (Olympus, Japan) was used for image capture.

### *In vitro* invasion assays

Cells (5 × 10^5^) were plated on the top side of apolycarbonate Transwell filter coated with Matrigel in the top chamber of the QCM 24-well cell invasion assay system (Cell Biolabs, Inc. Santiago, USA). The medium in the upper chamber was serum-free, and the serum-containing medium in the bottom chamber was used as a chemoattractant. After incubating the cells at 37 °C for 48 h, noninvasive cells in the upper chambers were discarded and the invaded cells on the bottom membrane surface were fixed in 100 % methanol for 10 min, air-dried, stained with crystal violet (Beyotime), and counted under a microscope. Three independent experiments were conducted.

### Animal experiments

To construct human bladder cancer xenograft models in nude mice, we purchased a total of 40 six-week-old female nude mice (BALB/c-nu/nu) weighing 16–20 g from SLAC Laboratory Animal Co. LTD. China (Changsha, China), which were randomly divided into the following 8 groups (5/group): A nontransfected J82 cell group (Vector-NC group), a Hsp27-overexpressing J82 cell group (Lv-Hsp27), a HMGN5-overexpressing J82 cell group (Lv-HMGN5), an Hsp27 + HMGN5 cotransfecting J82 cell group (Lv-Hsp27 + Lv-HMGN5), an empty plasmid transfected J82 cell group (Lv-sh-NC group), an Hsp27-silencing J82 cell group (Lv-sh-Hsp27), an HMGN5-silencing J82 cell group (Lv-sh-HMGN5), and an sh-Hsp27 + sh-HMGN5 cotransfecting J82 cell group (Lv-sh-Hsp27 + Lv-sh-HMGN5). Cell injections were performed as described previously [39] with the approval of The Third Xiangya Hospital of Central South University. The tumor volume and weight, and the protein levels of E-cadherin and Vimentin were examined in the indicated time.

### Statistical analysis

Data are expressed as the means ± SD of at least three independent experiments and statistically analyzed by one-way analysis of variance (ANOVA) followed by Tukey's multiple comparison tests or independent sample *t*-test using SPSS Statistics 17.0 software. The level of significance was based on the probability of *P* < 0.05 and *P* < 0.01.

### Ethics approval

The study was performed in accordance with the Declaration of Helsinki, and the praotocol was approved by the Ethics Committee of The Third Xiangya Hospital of the Central South University. All of the enrolled bladder cancer patients signed informed consent forms.

## Supplementary Material

Supplementary Figures

Supplementary Table 1

Supplementary Table 2
