# Supplementary material for: HMGN5 promotes IL-6-induced epithelial-mesenchymal transition of bladder cancer by interacting with Hsp27
Source: Aging (Albany NY). 2020 Apr 21;12(8):7282–98. doi: 10.18632/aging.103076 (PMC7202510; doi:10.18632/aging.103076)
Supplement: Supplementary Figures [file aging-12-103076-s003..pdf]

SUPPLEMENTARY FIGURES

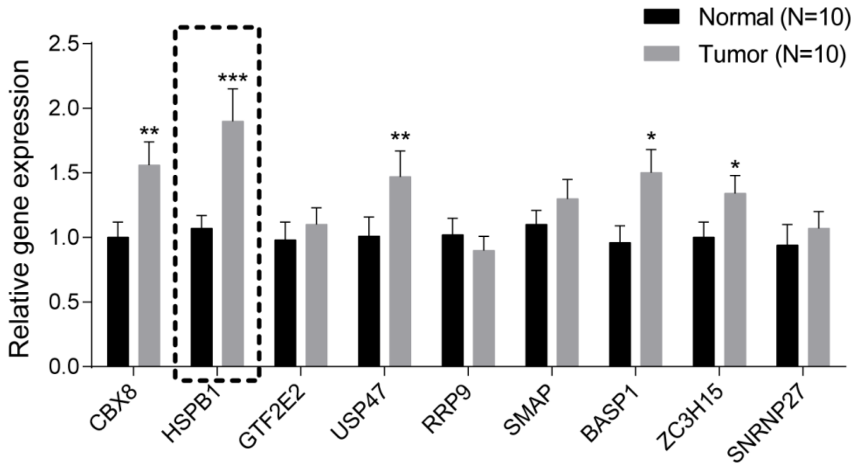

Supplementary Figure 1. The mRNA expression levels of the 9 genes (CBX8, HSPB1, GTF2E2, USP47, RRP9, SMAP, BASP1, ZC3H15, SNRNP27) in 10 bladder cancer tissues.

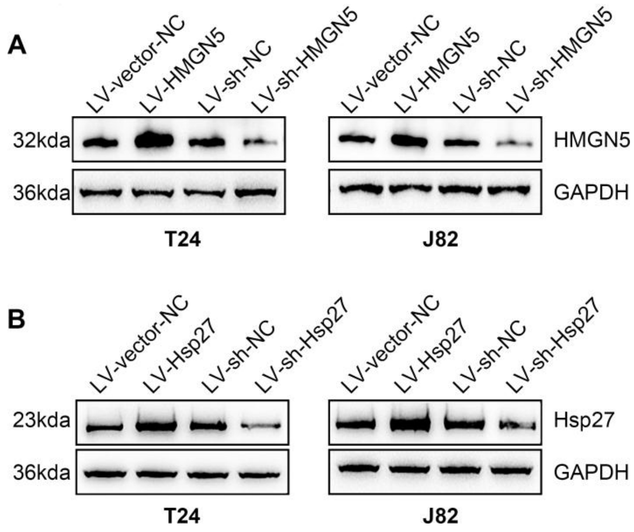

Supplementary Figure 2. The transduction efficiency of LV-HMGN5, LV-sh-HMGN5 (**A**), LV-Hsp27 and LV-sh-Hsp27 (**B**) in T24 and J82 cells as confirmed by immunoblotting analyses.
