# Supplementary material for: HMGN5 promotes IL-6-induced epithelial-mesenchymal transition of bladder cancer by interacting with Hsp27
Source: Aging (Albany NY). 2020 Apr 21;12(8):7282–98. doi: 10.18632/aging.103076 (PMC7202510; doi:10.18632/aging.103076)
Supplement: Supplementary Table 1 [file aging-12-103076-s001..docx]

**Supplementary Table 1. The clinical characterizations of bladder cancer patients.**

| Patient number | Hsp27 expression | Hsp27 level | HMGN5 expression | HMGN5 level | Time (mon) | Case | Age | Gender | Tumor grade | Tumor size | TNM stage | Tumor number |
| --- | --- | --- | --- | --- | --- | --- | --- | --- | --- | --- | --- | --- |
| 1 | 1.647 | Low | 1.055 | Low | 29 | 1 | 62 | M | 1 | ≥3 | III | multiple |
| 2 | 1.481 | Low | 1.666 | High | 60 | 0 | 32 | M | 1 | <3 | IV | multiple |
| 3 | 1.421 | Low | 1.423 | Low | 49 | 0 | 22 | M | 2 | ≥3 | I | single |
| 4 | 1.555 | Low | 1.769 | High | 58 | 0 | 61 | M | 1 | <3 | IV | single |
| 5 | 1.538 | Low | 2.31 | High | 60 | 0 | 67 | M | 2 | <3 | II | multiple |
| 6 | 1.557 | Low | 1.398 | Low | 60 | 0 | 34 | M | 1 | <3 | I | multiple |
| 7 | 1.541 | Low | 1.078 | High | 55 | 1 | 48 | F | 2 | ≥3 | III | single |
| 8 | 2.185 | High | 2.075 | High | 60 | 0 | 29 | M | 1 | <3 | IV | multiple |
| 9 | 1.422 | Low | 1.486 | Low | 60 | 0 | 27 | M | 1 | <3 | I | multiple |
| 10 | 1.66 | Low | 1.302 | Low | 60 | 0 | 27 | M | 1 | <3 | III | multiple |
| 11 | 1.896 | High | 2.085 | High | 60 | 0 | 54 | F | 1 | ≥3 | IV | multiple |
| 12 | 2.054 | High | 1.817 | High | 59 | 0 | 66 | M | 3 | <3 | IV | multiple |
| 13 | 1.25 | Low | 1.374 | Low | 60 | 0 | 59 | M | 1 | <3 | I | multiple |
| 14 | 1.737 | High | 2.125 | High | 16 | 1 | 35 | M | 1 | ≥3 | IV | multiple |
| 15 | 1.666 | High | 1.163 | Low | 60 | 0 | 60 | M | 2 | ≥3 | III | single |
| 16 | 1.745 | Low | 1.142 | Low | 52 | 0 | 44 | F | 1 | <3 | I | multiple |
| 17 | 1.232 | Low | 1.429 | Low | 60 | 0 | 46 | M | 1 | <3 | I | multiple |
| 18 | 0.661 | Low | 1.456 | Low | 60 | 0 | 70 | M | 1 | <3 | II | multiple |
| 19 | 1.801 | High | 1.703 | High | 31 | 1 | 65 | M | 1 | ≥3 | IV | multiple |
| 20 | 1.59 | Low | 1.962 | High | 60 | 0 | 65 | M | 2 | ≥3 | II | multiple |
| 21 | 1.513 | Low | 1.382 | Low | 60 | 0 | 73 | F | 2 | <3 | II | multiple |
| 22 | 1.597 | Low | 1.389 | Low | 55 | 1 | 63 | F | 2 | <3 | III | single |
| 23 | 2.075 | High | 1.816 | High | 35 | 1 | 48 | M | 3 | <3 | III | multiple |
| 24 | 1.998 | High | 0.615 | Low | 60 | 0 | 56 | M | 1 | <3 | I | multiple |
| 25 | 2.561 | High | 1.95 | High | 5 | 1 | 69 | M | 3 | <3 | III | single |
| 26 | 1.727 | High | 1.643 | High | 25 | 1 | 73 | M | 1 | ≥3 | IV | multiple |
| 27 | 1.335 | Low | 1.923 | High | 52 | 1 | 72 | F | 3 | ≥3 | III | multiple |
| 28 | 1.604 | Low | 2.285 | High | 60 | 0 | 55 | M | 1 | <3 | II | single |
| 29 | 1.059 | Low | 1.847 | High | 32 | 1 | 46 | M | 2 | <3 | II | multiple |
| 30 | 2.114 | High | 2.084 | High | 26 | 1 | 28 | M | 3 | <3 | III | multiple |
| 31 | 1.685 | High | 1.554 | Low | 36 | 1 | 61 | M | 2 | ≥3 | II | multiple |
| 32 | 1.775 | High | 1.859 | High | 24 | 1 | 52 | M | 1 | ≥3 | IV | multiple |
| 33 | 1.014 | High | 1.031 | Low | 47 | 1 | 22 | M | 2 | ≥3 | IV | single |
| 34 | 1.727 | High | 1.039 | Low | 60 | 0 | 47 | M | 2 | <3 | I | multiple |
| 35 | 2.158 | High | 2.322 | High | 14 | 1 | 48 | M | 1 | ≥3 | I | multiple |
| 36 | 2.15 | High | 2.226 | High | 6 | 1 | 38 | M | 3 | <3 | IV | multiple |
| 37 | 0.273 | Low | 0.777 | Low | 60 | 0 | 37 | M | 1 | ≥3 | I | single |
| 38 | 1.299 | Low | 0.981 | Low | 44 | 0 | 65 | M | 1 | <3 | I | multiple |
| 39 | 1.407 | Low | 2.33 | Low | 55 | 0 | 65 | M | 2 | ≥3 | I | multiple |
| 40 | 2.1 | High | 1.718 | High | 60 | 0 | 46 | M | 1 | ≥3 | III | single |
| 41 | 1.112 | Low | 1.436 | Low | 47 | 1 | 63 | M | 1 | <3 | I | multiple |
| 42 | 2.441 | High | 2.008 | High | 11 | 1 | 39 | M | 1 | ≥3 | III | single |
| 43 | 1.763 | High | 0.929 | Low | 60 | 0 | 41 | M | 2 | ≥3 | I | multiple |
| 44 | 1.663 | Low | 1.357 | Low | 60 | 0 | 44 | F | 3 | <3 | II | multiple |
| 45 | 2.009 | High | 1.518 | Low | 60 | 0 | 43 | F | 1 | <3 | IV | multiple |
| 46 | 1.899 | High | 1.694 | High | 60 | 0 | 48 | F | 2 | <3 | II | multiple |
| 47 | 2.14 | High | 1.741 | High | 22 | 1 | 61 | M | 1 | <3 | IV | single |
| 48 | 1.721 | High | 1.357 | Low | 60 | 0 | 58 | F | 2 | <3 | II | multiple |
| 49 | 1.088 | Low | 0.999 | Low | 60 | 0 | 39 | M | 2 | <3 | I | multiple |
| 50 | 1.554 | Low | 1.635 | High | 60 | 0 | 52 | M | 2 | ≥3 | I | multiple |
| 51 | 1.836 | High | 1.441 | Low | 37 | 1 | 51 | F | 1 | <3 | II | single |
| 52 | 1.844 | High | 0.896 | Low | 60 | 0 | 48 | F | 2 | ≥3 | II | multiple |
| 53 | 1.731 | High | 1.976 | High | 17 | 1 | 69 | M | 3 | ≥3 | IV | multiple |
| 54 | 1.835 | High | 2.112 | High | 42 | 1 | 69 | M | 1 | <3 | IV | single |
| 55 | 1.452 | Low | 1.408 | Low | 56 | 1 | 39 | M | 3 | ≥3 | IV | multiple |
| 56 | 1.406 | Low | 1.745 | High | 60 | 0 | 65 | M | 2 | <3 | II | single |
