# Supplementary material for: HMGN5 promotes IL-6-induced epithelial-mesenchymal transition of bladder cancer by interacting with Hsp27
Source: Aging (Albany NY). 2020 Apr 21;12(8):7282–98. doi: 10.18632/aging.103076 (PMC7202510; doi:10.18632/aging.103076)
Supplement: Supplementary Table 2 [file aging-12-103076-s002..docx]

Supplementary Table 2. Univariate and multivariate analysis for factors related to overall survival using the COX proportional hazard model.

| **Characteristics** | | **Univariate analysis** | | |  | **Multivariate analysis** | | |
| --- | --- | --- | --- | --- | --- | --- | --- | --- |
|  |  | **P** | **HR** | **95.0% CI** |  | **P** | **HR** | **95.0% CI** |
| Hsp27 | High vs Low | .008 | 3.364 | 1.379-8.207 |  | .084 | 2.331 | 0.894-6.076 |
| HMGN5 | High vs Low | .009 | 3.265 | 1.339-7.957 |  | .073 | 2.355 | 0.922-6.015 |
| Age | 51.18±14.21 | .426 | 1.012 | 0.982-1.044 |  |  |  |  |
| Gender | Female vs male | .359 | .603 | 0.205-1.776 |  |  |  |  |
| Tumor grade | 1 vs 2&3 | .990 | 0.995 | 0.439-2.257 |  |  |  |  |
| Tumor size | <3 vs ≥3 | .057 | .448 | 0.196-1.023 |  |  |  |  |
| TNM stage | I + II vs III + IV | .005 | .280 | 0.115-0.682 |  | .147 | .481 | 0.179-1.293 |
| Tumor number | Multiple vs Single | .323 | .648 | 0.275-1.531 |  |  |  |  |
